# Role of novel policy implementation for Gamma Knife (GK) procedures in improving access to neurosurgical care in lower middle income countries (LMICs) GK in LMICs

**DOI:** 10.1016/j.wnsx.2023.100166

**Published:** 2023-01-31

**Authors:** Bhavya Pahwa, Deepak Agrawal

**Affiliations:** a Medical Student, University College of Medical Sciences, Delhi, India; b Department of Neurosurgery, All India Institute of Medical Sciences, New Delhi, India

**Keywords:** Gamma knife radiosurgery, Lower middle income countries, MRI, Arteriovenous malformations (AVMs), Gamma knife (GK), International Atomic Energy Agency (IAEA), Lower middle income countries (LMICs), Magnetic resonance imaging (MRI), Operating room (OR)

## Abstract

**Background:**

The high cost of GK machines with resultant scarcity of GK centers, coupled to additional cost of contrast MRI brain makes it nearly impossible for the vast majority of the population without health insurance in LMICs to have access to this advancement in management.

**Methods:**

AIIMS, Delhi is the premier public funded hospital in India with the largest Neurosurgical department in the country. We implemented a single payment policy which would cover the treatment and all subsequent GK treatments as well as Follow-up MRIs of the patient. The program was named ‘Gamma Knife for life’ and implemented in 2015. To check the efficacy, we conducted a retrospective analysis of the Neurosurgery cases managed before and after the implementation of this scheme in the last 12 years.

**Results:**

Prior to implementation of the ‘Gamma Knife for life’ program, a total of 24,703 patients underwent Neurosurgical procedures in the years 2009–2015. Of these, only 2017 (8.16%) were GK procedures. In the years following the introduction of the program, the total number of neurosurgical procedures was 23,030 with 2947 (12.8%) being the GK procedures This difference in the number of GK procedures before and after the application of ‘Gamma Knife for Life’ was statistically significant (p = 0.026). The highest number of GK procedures (n = 789) reported so far were in 2020–21.

**Conclusions:**

Ensuring holistic care by providing for not only the initial GK procedure but all subsequent procedures, as well as follow-up MRI's can improve the quality and consistency of care, especially in LMICs.

## 1 Introduction

Every 1 million Indian population is served by only one neurosurgeon, when ideally, there should be at least one neurosurgeon per 0.1 million population.^1^ There is an unprecedented imbalance in the number of patients and the neurosurgeons available to manage them in india. Hence, the vast majority of the population goes without getting adequate treatment and rather no treatment at all.

Recent years have seen an uprise in the utilization of Gamma Knife (GK) in neurosurgical practice. Being minimally invasive, having minimal side effects and a high efficacy, GK is now being used as a primary as well as an adjuvant treatment for a multitude of neurosurgical pathologies.^2^ However, this advancement in medical science is not without limitations, especially in Lower Middle Income countries (LMICs) like India and Africa. An Indian family survives on an average monthly income of $100 while the average cost of GK treatment in India in the private sector is $6500 which is 650 times the income.^3^ In public funded hospitals, the cost of one GK session is still $1000. Since most of the population is uninsured, there is a tendency for them to not show up for another GK appointment or to not undergo GK. There are only 6 functional GK centers in India^4^ and that too in developed cities. Additionally, there is a need for contrast Magnetic Resonance Imaging (MRI) almost throughout life at least on a two yearly basis which adds on to the treatment cost.

On the other hand, due to heterogeneous quality of Neurosurgical training across different hospitals, there is lack of surgical consistency and patient outcome across different centers. Follow up is also lacking due to poor socio-economic status of most of the treated population. Due to very few centers doing complex Neurosurgical cases, waiting times for surgery can be very long, extending to over two years in our institute!

The current paucity of personnel and infrastructure to build and operate GK centers calls for new policies that could help deliver GK neurosurgical care in LMICs with limited resources. A paradigm policy shift with increase in Gamma-knife installations along with telemedicine, remote planning and treatment delivery may transform Neurosurgical healthcare delivery for India's 1.4 billion population. This study aims at studying the efficacy of a pilot programme (named“*Gamma Knife for Life”)* at a tertiary care hospital in India *.*

## 2 Material and methods

### 2.1 Strategy

This pilot program was implemented in a public funded research and educational hospital in India: All India Institute of Medical Sciences (AIIMS), New Delhi in April 2015. Use was made of already available resources like Gamma Knife machine (Elekta^Ⓡ^, Stockholm, Sweden), MRI machine, medications and other required medical stocks. Under this policy, the patients with conditions that were considered to be suitable for GK as per neurosurgeon's discretion were enrolled in this programme, irrespective of their financial status. A Gamma knife ICON^Ⓡ^ machine costs around 3 million USD in India (Including 10 year maintenance contract). This cost is borne by the hospital (through public funds). Source reloading is normally done every 5 years and costs another one million USD and is paid through the charges collected from patients undergoing Gamma knife. Patients who were insured or if their healthcare finances were covered under any government programme, were automatically enrolled under this initiative named ‘GK for life’. Following enrollment, patients were asked to pay the existing charges of $1000 (INR 75000) upfront as their entire treatment fee that included GK procedures and *all* follow up MRIs throughout their life. Pathologies included brain tumors (Metastasis, Acoustic Neuromas, schwannomas, Meningiomas, Pituitary tumors, Craniopharyngiomas, hemangioblastomas, glomus jugulare tumors and gliomas), vascular malformations (AVM's, Cavernomas & DAVF's) and functional pathologies like trigeminal Neuralgia & hemifacial spasm. This was a retrospective analysis wherein records of patients managed with GK (primary or secondary) five years before and six years after the implementation of the programme were searched and the total number of patients treated annually was recorded. Hence, this was a 11 year long study starting from 2009 till 2021.

### 2.2 Statistical analysis

Statistical analysis was performed using the SPSS software (version 13.0) to evaluate the efficacy of this programme. Student's *t* test was utilized to compare the means of the two groups. *p* value of <0.05 was considered statistically significant.

## 3 Results

### 3.1 Before the implementation of ‘GK for life’

Prior to the implementation of the programme, 24,703 neurosurgical procedures (including GKRS) were performed at our center over a five year period, with maximum being performed in 2012–13 (n = 6234, 37%). Of these 2017 (8.16%) patients underwent GK with the majority of them in the year 2011–12 (n = 588, 29.15%).

Data was not available for the year 2009–10. In 2010–11, 3966 patients were treated (209 with GK). In 2011–12, 3859 were treated (588 with GK). A total of 6234 patients underwent neurosurgical procedures in 2012–13 of which 398 (6.38%) were GK procedures. In 2013–14, 375 (7.09%) GK procedures were performed out of the 5286 neurosurgical procedures. Just before the implementation of our programme in April 2015, 5358 patients were neurosurgically treated at our center from 2014 to 2015 of which 447 (8.34%) were treated with GK.

**Fig. 1:**
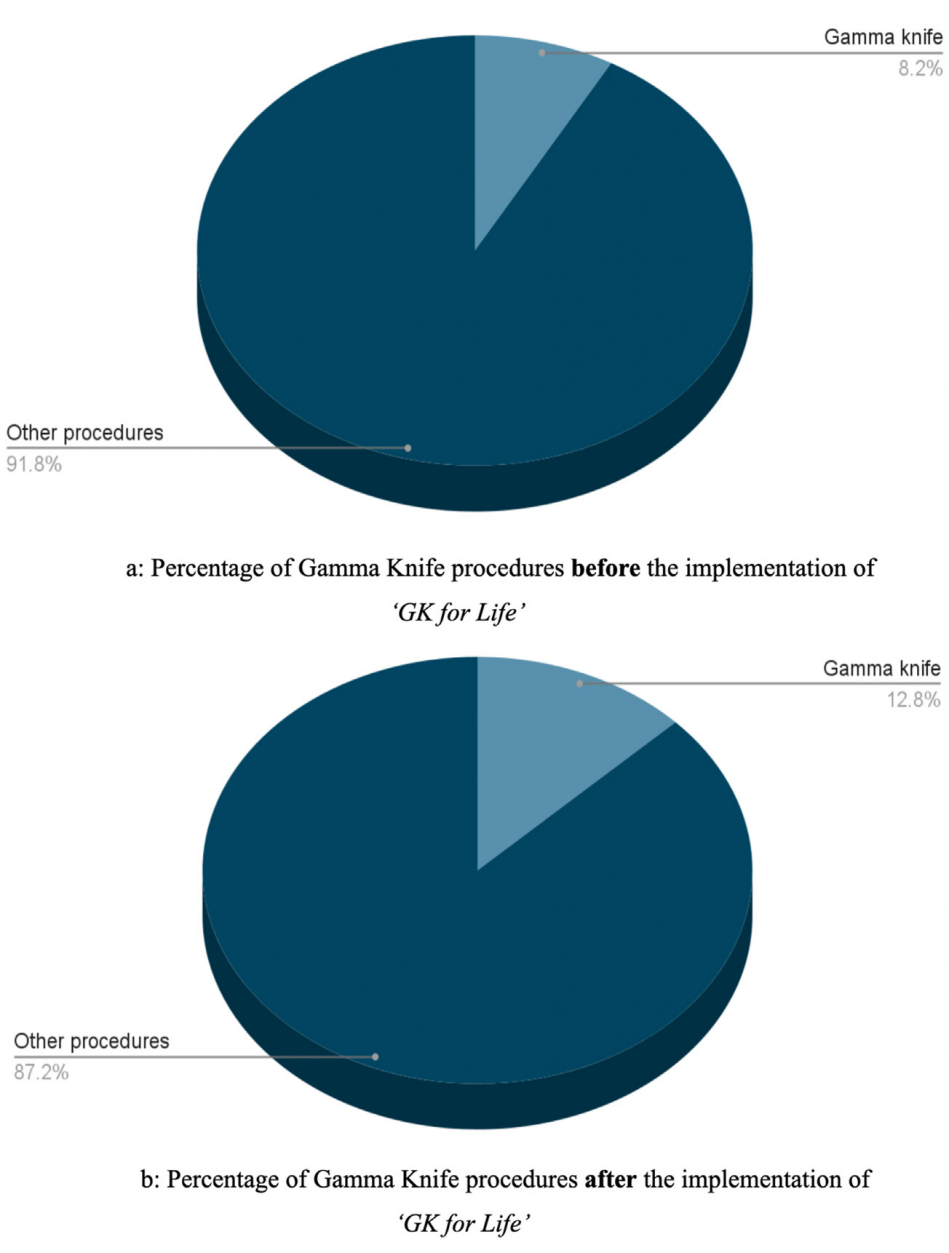
Comparison between Gamma knife procedures carried out at our center before and after the application of ‘GK for Life’. Fig. 1a: Percentage of Gamma Knife procedures **before** the implementation of *‘GK for Life’.*Fig. 1b: Percentage of Gamma Knife procedures **after** the implementation of *‘GK for Life’*.

Fig. 1 depicts the percentage of GK procedures before (Fig. 1a) and after (Fig. 1b) the implementation of ‘GK for life’.

### 3.2 After the implementation of ‘GK for life’

For the six years following the implementation of *‘GK for life’,* 23,030 patients were neurosurgical treated (including GK) for various pathologies with maximum patient load in the year 2016–17 (n = 5572, 24.2%). There was a substantial rise in the percentage of GK procedures performed after the implementation of *‘GK for life’*. A total of 2947 (12.8%) GKRS procedures were performed post ‘*GK for life’* scheme of which the pandemic year, 2020–21 reported maximum number of procedures till date (n = 789, 26.78%).

**Fig. 2:**
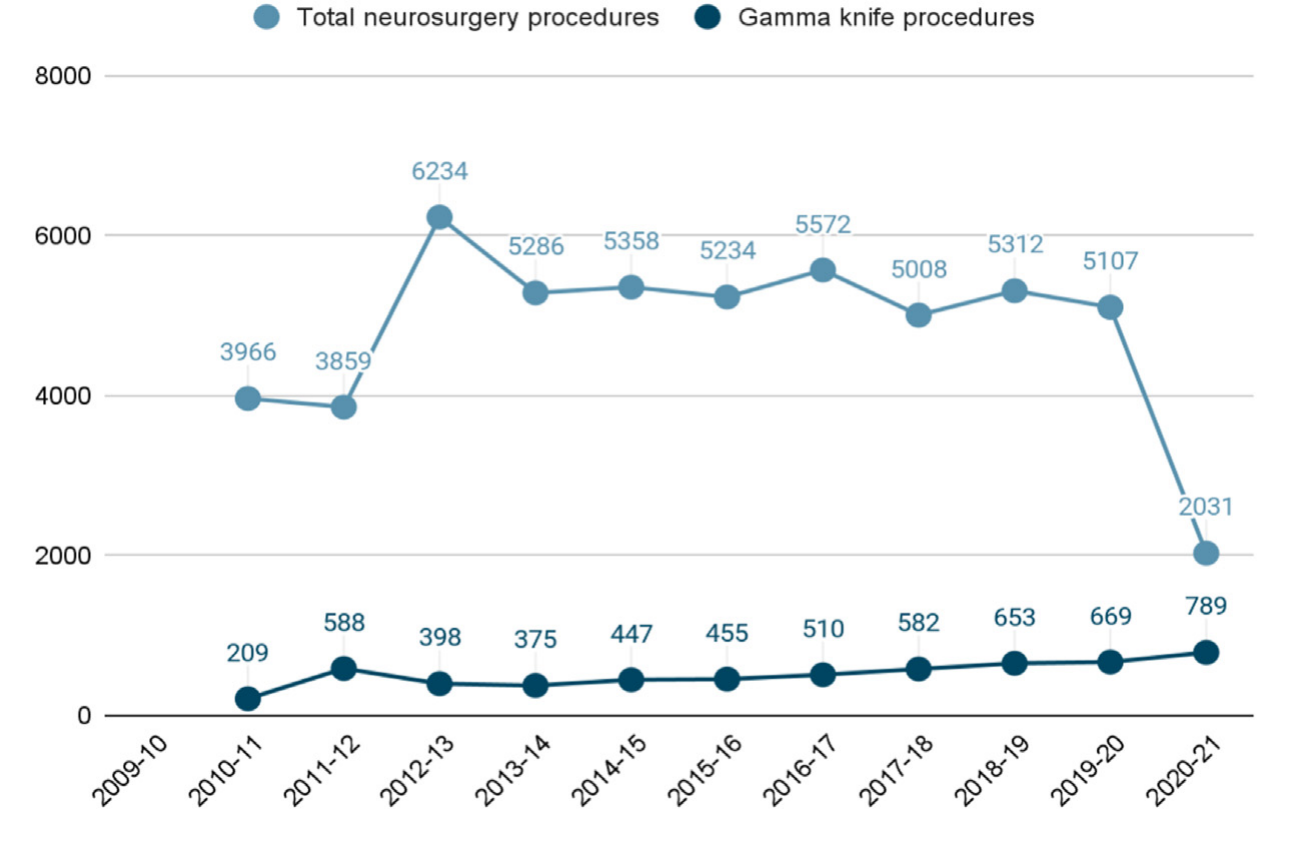
Trends in the number of total neurosurgical procedures and Gamma knife procedures carried out at our center in the last decade.

The number of total neurosurgical procedures performed remained almost comparable except in the year 2020–21 owing to COVID-19 pandemic when the number of total neurosurgical procedures decreased drastically (n = 2031). However, the frequency of GK procedures increased over the years (Fig. 2).

The mean number of GK procedures performed before the implementation of the program was 403 while after implementing the programme, a mean of 610 GK procedures were done every year. This difference was statistically significant **(p** = **0.026).** The rate of change in the number of GK cases per year after the introduction of this policy was significantly higher than that before (55.67% vs 39.67%, **p** = **0.0001**). However, the difference in the mean number of percentage of GK procedures performed per year before the implementation and after the implementation was not statistically significant (7.05% vs 15.6%, p = 0.128).

### 3.3 Estimated life-cycle cost savings

A Gamma knife ICON^Ⓡ^ machine costs around 3 million USD in India (Including a 10 year maintenance contract). Source reloading costs another million USD and is normally done every 5 years. All staff are on hospital payroll with the total cost being around $340000/year (Table 1). The current cost of one GK procedure at our center is $1000 and the current cost of contrast MRI is $50. Under the *‘Gamma Knife for Life’* program, the patient undergoes the GK procedure (one or more) and all the follow up MRIs for $1000. As per the current policy of one yearly MRI for 2 years followed by every 2 years for life with a mean follow up of 15 years, there is saving of $800 to each patient, not including inflation and interest. The number of patients undergoing repeat GK was less than 10%, however the exact number was not available, hence the actual saving is more than $800 per patient.

**Table 1:** Staff costing for a typical Gamma Knife ICON centre in India working in 2 shifts/day (8am-8pm) averaging 1500 GK procedures every year.

Staff	Salary USD/year	Number required	Total cost USD/year
Neurosurgeon	100,000	2	200,000
Physicist	15,000	2	30,000
Nurses	7500	4	30,000
Radiation workers	7500	4	30,000
Other staff	5000	10	50,000
			**340,000**

We have recently upgraded our machine to Gamma Knife ICON^Ⓡ^ and the patient throughput has increased dramatically to 120 patients/month. We anticipate performing around 1500 GK procedures this year. Using this patient volume, our revenue will be $1.5 million every year and expenditure is calculated at $385 k (Table 2), leading to net revenue of $3.55 million over a 10 year period (Table 3).

**Table 2:** Expenditure for a typical Gamma Knife ICON centre in India working in 2 shifts/day (8am-8pm) averaging 1500 GK procedures every year.

Head	USD/year	Cost over 10 years
Staff	340,000	$3.4 million
CMRI @$50	75,000	$3.75 million^a^
Electricity etc.	30,000	$0.3 million
Total	**385,000**	**$7.45 million**

a We have assumed 5 MRI for each patient for a total of 15,000 patients over a 10 year period).

**Table 3:** Life. Cycle costing for a Gamma knife machine in India over 10 years with a medium case load (1200 patients/year) and high case load (1500 patients/year).

Head	USD/year	Cost over 10 Years
Revenue @1500 patients/year	15,000,000	$15 million
Revenue @ 1200 patients/year	10,000,000	$12 million
Capital Expenditure (GK machine)	30,000,000	$ 3 million
Other Expenditure (see table y)	385,000	$7.45 million
Source reloading	1,000,000	$1 million
Net revenue @1500 patients/year		**3.55 million**
Net revenue @ 1200 patients/year		**0.55 million**

## 4 Discussion

GK is a very safe and efficacious treatment modality for a kaleidoscope of neurosurgical pathologies including intracranial metastasis, meningiomas, vestibular schwannomas, trigeminal neuralgia and arteriovenous malformations (AVMs). It does not require an operating room (OR) and is mostly performed under local anesthesia (some pediatric cases might require general anesthesia) in the outpatient department only. Unlike invasive surgeries, it does not require a large group of personnel for performing the procedure which significantly decreases the healthcare burden. Therefore, GK can be a boon for countries with excessive patient load and paucity of resources and infrastructure. Neurosurgical pathologies like AVMs can be managed with open surgery, GK and/or interventional neuroradiology (embolisation). GK has been proven to be a safe and efficacious alternative to the other two techniques mentioned^5^, thereby decreasing burden on them and making them available for other pathologies that cannot be managed with GK. This ensures an improved neurosurgical throughput and consistency in care.

However, the reach of this technology is largely limited to the developed world like , the United States, Europe, Canada and some parts of Asia, while Africa, South America and a large part of Asia are deprived of adequate GK facilities. Since GK is non invasive, highly efficient, especially in areas which are economically non-compliant to open surgeries, it is imperative to introduce GK in socioeconomic backward countries to aid in better neurosurgical decision making. Fezeu et al^6^ reported for the first time, the global disparities pertaining to accessibility of GK wherein they identified that developed nations had approximately 1 GK center per 3 million population as compared to 1 GK center for over a hundred million population in developing nations. Pannullo et al^7^ reported extensive geographical variations in radiosurgical accessibility with nearly 10 million people per device in North America, Europe and Australia to one device per 35 million in South America and only 1 device per one billion population in the African continent. There are only 6 GK centers in India catering to a population of over 1 billion with approximately 1 device per 143 million people. There are a multitude of challenges for expanding access to GK in India and other developing countries.

Dearth of reliable disease data recording and management systems and limited medical resources in LMICs make it difficult to identify the patients who would otherwise be fit for GK. Additionally, a GK repository like ours aids in monitoring the treatment response to GK while facilitating the neurosurgeons to have a reference for dosimetric analysis and future research purposes. Also, most tumors and vascular pathologies are amenable to radiosurgery and preferentially providing GK to these patients significantly decreases the surgical load on the Neurosurgeons. Importantly, as both GK and surgery is performed by Neurosurgeons, treatment decisions are more balanced and ‘patient centric’ potentially resulting in better outcomes. Prior to this program, patients not affording GK were put in the waiting list for surgery which already exceeds 2 years (at our institute)! This results in delayed/suboptimal treatment to the majority of the patients. Also, as follow up imaging is not free, most patients could not afford the CMRI brain charges and were lost on follow up. After implementing this program, all patients are guaranteed free follow up imaging and treatment for life with follow up rates now close to 100% ensuring better outcome analysis, which in turn lead to improved policy planning and resource allocation.

Performing a GK procedure requires a team approach including a neurosurgeon, radiation oncologist, physicist and a nurse. There is a paucity of trained neurosurgical personnel in LMICs with one neurosurgeon per 6,400,000 patients.^8^^,^^9^^,^ Nearly 5 million of the treatable ‘essential neurosurgical cases’ never get the required care, with majority of them belonging to Africa and Southeast Asia.^10^ Additionally, there is greater burden of neurosurgical diseases in the developing world which necessitates the neurosurgeons in these countries to get trained in minimally invasive and ‘non essential’ sub specialities like stereotactic and functional neurosurgery.^7^ We therefore need to optimize this existing skilled manpower and here Gamma knife fits perfectly by providing ‘last mile connectivity’ for the patients. Using Telemedicine with remote diagnosis along with remote GK planning we can revolutionize neurosurgical healthcare delivery with the existing resources. Follow-up can also be taken care at the local GK center with only those patients requiring surgical intervention being referred to centrally located tertiary care centers.

### 4.1 Economic insufficiency

One of the biggest obstacles to the implementation of GK in LMICs is the high cost of machine installment and lack of infrastructure. This is worsened by the attached expenses of periodic maintenance of the machines. GK has a longer life (20 years) than LINAC (10 years) provided there is periodic repair and maintenance of the machine parts and resources. In the US, the average cost of radiosurgery ($35,734) is far lower as compared to open surgery ($67,270) for various neurosurgical pathologies including brain metastasis, vestibular schwannoma and AVMs.^11^ Though these are US estimates, we predict it to be similar in an Indian set up. As is seen from our life cycle cost analysis, investing in scores of GK installations (each attached to a Neurosurgical center), across India can be both economically viable over the long term besides providing consistent and high quality Neurosurgical care across the length and breadth of our country. We suggest a one time investment could be made using policies like ours that have the potential to ***immediately*** decrease the neurosurgical mortality and morbidity across the population.

Over the years, with better acceptance of Gamma knife and its indications amongst the Neurosurgical community, there has been a gradual increase in the number of patients being referred for GK. In our institute, especially after the implementation of the *‘GK for Life’* strategy, the GK numbers have dramatically increased, thereby paving a way for other centers in the country to adopt this strategy. The highest percentage of GK procedures was reported in the year 2020–21, which was also the year of the ongoing COVID-19 pandemic. That year saw a drastic fall in the number of Operation Room (OR) based surgeries and a paradigm shift to GK. Hence, GKRS proved to be an instrumental modality for both the patients and the providers in the pandemic times over microsurgery. Therefore in wake of unpredictable years ahead, we suggest an active installment of strategies and policies to enhance the access of GK in various parts of India and other developing countries especially because there has been a scarcity of the resources post 3rd wave of COVID-19 in most countries. GK being minimally labor and resource intensive holds immense potential to be an alternative to the current OR based surgeries.

### 4.2 Limitations

Many patients especially those with vascular lesions like AVMs are suitable for treatment with either surgery, GK or Embolisation (or a combination of these) and specifically funneling these patients towards GK frees up the other scarce treatment modalities for other patients. However, we don't have the exact number of such patients but in our experience many patients with AVMs started preferring GKRS over surgery. Also, we could not present the data for patients who would not otherwise have gotten GK treatment if it wasn't for the new policy. This could have provided more supportive evidence for our study.

#### 4.2.1 Future prospects, advancements and suggestions

Addition of technologies and better policies could facilitate an expansion of the already present GK centers to cater to larger patient size. GK usually involves placement of a stereotaxy frame which is known to cause anxiety in patients,^12^ requires proper sterilization and needs intensive resources. This can be overcome with the use of mask based immobilization which are relatively easier to put together.^13^, ^14^, ^15^ A part of this techniques can be automated with the help of 3 dimensional printing, simplifying the manufacturing of masks.^6^ The scarcity in the expert personnel can be overcame by the remote support of the neurosurgeons, radiation oncologists or therapists, thereby opening the possibility of delivering GKRS by less trained locally present staff. For instance, a 3 tier teleradiotherapy network model by Datta and Rajasekar^16^ has been extensively implemented by the Indian ministry of Health in various institutions. Similar model can be utilized while delivering GK but with the addition of a highly qualified neurosurgeon offsite. Policies like these will however, require extensive training which creates another challenge for the LMICs.

Foremost measure in expanding access of GK is to spread awareness amongst the masses regarding the utilization and efficiency of GK in the management of neurosurgical pathologies. Transcending the economic insufficiency will require funding from local governments and organizations that aim at globalizing the healthcare facilities. For instance, local governments in Africa are supported by the International Atomic Energy Agency (IAEA) for facilitating oncologic care and radiotherapy. IAEA provides training sessions and educational workshops to train healthcare providers in radiation oncology, thereby surpassing the shortage of trained personnel. Moreover, IAEA has also facilitated the development of infrastructure, availability of equipment and resources and appointing the required staff members. Additionally, radiosurgery societies around the globe have taken certain measures to improve and expand the access to GK. ‘GK for life’ program implementation will go a long way in making care more accessible and consistent. However, to make it a reality the cost of setting up a GK center should come down dramatically. There are now GK machines being manufactured by other vendors which are less than one-third the price of those made by Elekta.^17^ Although we have no experience of using them, we believe that a healthy competition in this sector may pave the way for deploying GK on a large scale across India and other developing countries. Finally, more policies like *“GK for Life”* can be implemented around the globe, thereby enhancing the reach of GK to otherwise impossible areas.

## 5 Conclusion

Lower and middle income countries (LMICs) are at a disadvantage owing to a highly distorted ratio of the patient load and the number of neurosurgeons. Additionally, scarcity of resources and the unstable economic condition of a vast majority of the population add on to the severity of the condition. GKRS has evolved as a potent treatment of a number of neurosurgical entities, however, its availability is largely limited to the developed world and a few developing nations. Strategies like *‘GK for Life’* pave a path for expanding the access to GKRS and aiding in establishing more GKRS centers throughout the world.

## Credit author statement

Study concept: BP. Study design: DA, Patient data collection: BP. Statistical analysis: BP. Manuscript writing: BP. Manuscript editing: DA.

## Funding

This research did not receive any specific grant from funding agencies in the public, commercial, or not-for-profit sectors.

## Declaration of competing interest

The authors declare that they have no known competing financial interests or personal relationships that could have appeared to influence the work reported in this paper.
